# Effect of Intraoperative Blood Pressure Regulation on Postoperative Hemorrhage After Bariatric Surgery

**DOI:** 10.1007/s11695-024-07275-5

**Published:** 2024-05-20

**Authors:** Mira Fink, Shayda Stock, Jodok Matthias Fink, Gabriel Seifert, Veit Broghammer, Stephan Herrmann, Stefan Fichtner-Feigl, Goran Marjanovic, Claudia Laessle

**Affiliations:** 1 Department of General and Visceral Surgery, Faculty of Medicine, Medical Center – University of Freiburg, Freiburg, Germany; 2 EXCEL Excellent Clinician Scientist Program, Else Kroener Research Schools for Physicians, Faculty of Medicine, University of Freiburg, Freiburg, Germany

**Keywords:** Blood pressure test, Postoperative hemorrhage, Bariatric surgery

## Abstract

**Introduction:**

With a rising number of bariatric procedures, the absolute number of postoperative complications is increasing, too. Postoperative bleeding, particularly along the staple line, is a recognized challenge. Numerous strategies including reinforcement of the staple line (SLR) have been proposed to improve bleeding rates, but no single technique has shown superiority over the others. In our bariatric center, we have implemented intraoperative blood pressure regulation alone, without SLR, to reduce hemorrhagic complications postoperatively.

**Methods:**

This retrospective observational analysis compares the incidence of postoperative bleeding in two groups of consecutive patients (total *n* = 438 patients), one with and one without intraoperative blood pressure elevation to 150 mmHg systolic without the additional use of staple line reinforcement. This intervention was integrated into our standard bariatric surgical procedure, no randomization or matching was conducted. Significant postoperative bleeding was defined as drop of hemoglobin of more than 2.5 mg/dl in 48 h and one of the following symptoms: lactate ≥ 2 mmol/L, bloody drainage, quantity of drainage more than 200 ml and/or radiological signs.

**Results:**

Defined postoperative bleeding occurred in 33 (7.5%) patients. We observed a decrease in bleeding rates from 10% to 5% (*n* = 22 vs. *n* = 11) after introducing intraoperative blood pressure increase (*p* = 0.034). The rate of revisional surgery for bleeding also decreased from 2.7% to 0.5% (*n* = 6 vs. *n* = 1). In multivariate analysis, the intervention with blood pressure elevation showed a significant decrease on bleeding rates (*p* = 0.038).

**Conclusion:**

The use of increased intraoperative blood pressure alone, without staple line reinforcement, appears to be an effective and suitable method for reducing post-bariatric hemorrhagic complications.

**Graphical Abstract:**

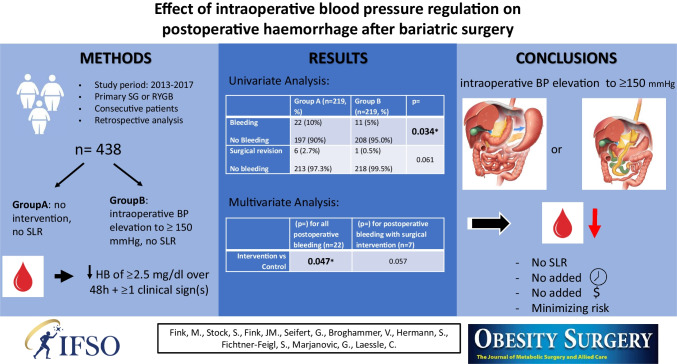

## Introduction

Bariatric surgery remains the most important pillar in the treatment of patients suffering from obesity and its relevant comorbidities. In 2022 a total of over 300,000 bariatric surgeries were performed worldwide [1]. As the number of patients with obesity is increasing steadily, the numbers of surgeries are predicted to continue to rise. Multiple long-term studies and reports have focused on the safety, morbidity, and mortality of bariatric surgery [2–8]. Although morbidity (up to 8%) and mortality rates (0.03–0.2%) are low, surgeons are continuously improving surgical techniques of bariatric procedures [8–10].

Perioperative bleeding, most commonly from the staple line, is a well-known complication after bariatric surgery. Rates up to 4% are seen after sleeve gastrectomy (SG) [11–18], with a similar incidence after Roux-Y-gastric bypass (RYGB) [19–22]. Treatment approaches include conservative treatment, endoscopic interventions, and revisional surgery (up to 50%). Higher 30-day mortality, longer hospital stay, and higher costs are encountered [15, 18, 19]. Multiple studies have shown that staple line reinforcements (SLR) can reduce the risk of early postoperative bleeding [11, 13, 17, 23]. A risk calculator (SLEEVE BLEED) showed that SLR and the level of expertise are independent predictors for postoperative bleeding after SG. Thus, SLR is recommended in the American guidelines [14, 24]. Different techniques including oversewing of the entire staple line, fibrin glue, polymer membranes, or special stapler devices have been tried and tested, but no single technique has been identified as superior. Despite current recommendations, multiple studies exist, which cannot show a significant improvement in bleeding rates with SLR alone [16, 25–28]. Thus, further intraoperative interventions were tested to minimize the risks of postoperative bleeding, including intraoperative blood pressure regulation. De Angelis et al. evaluated intraoperative blood pressure increase in combination with a decrease of the pneumoperitoneum in addition to SLR, which reduced bleeding rates from 1.7% to 0% [12]. Sroka et al. combined different SLR techniques with intraoperative systolic blood pressure increase to a minimum of 140 mmHg, which also showed to reduce bleeding from 9.5% to 3% [29]. There is, however, no evidence about the effectiveness of intraoperative blood pressure increase, alone, without SLR. As the use of SLR has additional material costs and significantly longer operating times, our bariatric center has only used intraoperative blood pressure regulation to decrease hemorrhagic complications after bariatric surgery, with promising clinical results. This retrospective analysis compares the rate of postoperative bleeding after bariatric surgery for a group of consecutive patients with and without intraoperative blood pressure regulation.

## Methods

This monocenter retrospective observational analysis included all consecutive patients who underwent laparoscopic bariatric surgery from January 2013 to May 2017 according to German S3 guidelines [30]. Included were both SG and RYGB. All patients taking therapeutic anticoagulation preoperatively were operated under prophylactic anticoagulation and received standard prophylactic doses of anticoagulation postoperatively. No patients had an indication for continued used of therapeutic anticoagulation intraoperatively or immediately postoperative. Informed consent was obtained for all patients. Since March 2015, we revised our surgical technique and all patients underwent intraoperative blood pressure increase to ≥ 150 mmHg systolic pressure with selective clipping of the staple line. Thus, all patients prior to March 2015 were included in the control group (group A), and all patients receiving intraoperative blood pressure regulation after March 2015 were included in group B (intervention). For neither group SLR was performed. As this was an observational retrospective study, no matching patient parameters between groups was performed. All procedures performed in studies involving human participants were in accordance with the ethical standards of the institutional research committee and with the 1964 Helsinki declaration and its later amendments or comparable ethical standards. Informed consent was obtained from all individual participants included in the study.

All operations were performed laparoscopically by the same team of experienced (more than 5 years) bariatric surgeons. A KLS Martin *MarSEAL* device was used for tissue dissection and Medtronic *EndoGIA stapler* (45–60 mm purple) to form the sleeve/pouch. The intraabdominal pressure was kept at 15 mmHg throughout the entire operation, a change was not routinely performed. The increase of systolic blood pressure was regulated at the end of resection by the anesthesiologist, who gave catecholamines aiming for a systolic pressure of ≥ 150 mmHg. No side-effects of this intervention were observed. Staple line bleeding could be easily identified, and titanium clips were used selectively to stop bleeding points. No further staple line reinforcement was used. In all cases, abdominal drainage was placed for the first 24 h postoperatively.

For the purpose of this study, postoperative bleeding was defined as a drop in hemoglobin of ≥ 2.5 mg/dl over 48 h and one or more of the following clinical signs: lactate ≥ 2 mmol/L, bloody drainage, quantity of drainage more than 200 ml and/or radiological signs of bleeding (free fluid in ultrasound or computer tomography). The indication for emergency revisional surgery was based on the presence of free fluid, cardiovascular instability (with the need for catecholamines postoperatively), or continued drop in hemoglobin.

Patient data including demographics, body compositions, comorbidities, medication, and surgical and anesthesiologic data were extracted from electronic patient charts.

Statistical analysis was performed using SPSS 29.0 (SPSS Inc.). Descriptive data was given for nominal and ordinal variables. Univariate analysis was performed with Student’s *T*-test for continuous variables and either Pearson’s chi square or Fisher’s exact test for categorical variables. Multivariate analysis was performed with a confidence interval at 95%. Significance level was set at *p* < 0.05.

## Results

### Patient Demographics

Overall, 438 patients were included (group A *n* = 219, group B *n* = 219). 78.3% were females and 21.7% were males (*n* = 343 and *n* = 95, respectively *p* = 0.052). Preoperative weight (138.5 kg ± 24.3) and BMI (48.6 kg/m2 ± 7.4) were higher in control group (group A) compared to intervention group (group B) (130.4 kg ± 25.8, 45.6 kg/m2 ± 7.3, both *p* < 0.001). More SGs were performed in group A (*n* = 147 vs. *n* = 74), whereas more RYGB were performed in group B (*n* = 72 vs. *n* = 145, *p* < 0.001). Except for selective clipping of the staple line during blood pressure increase, no further surgical intervention was performed.

Besides body weight, hypertension and surgery type all examined parameters and patient demographics were equally distributed between groups, depicted in Table 1.

**Table 1:** Patient demographics for group A (control) and group B (intervention)

	Control group A (*n* = 219, %)	Intervention group B (*n* = 219, %)	Overall (*n* = 438, %)	Significance (*p* =)
Sex				0.052
Male	55 (25.1%)	40 (18.3%)	95 (21.7%)	
Female	164 (74.9%)	179 (81.7%)	343 (78.3%)	
Age (mean, SD)	44.4 ± 11.4	42.6 ± 11.9	43.5 ± 11.7	0.058
Pre-op weight (kg)	138.5 ± 24.3	130.4 ± 25.8	134.4 ± 25.3	< 0.001 *
Pre-op BMI (kg/m^2^)	48.6 ± 7.4	45.6 ± 7.3	47.1 ± 7.5	< 0.001*
ASA classification				0.159
1	3	0	3	
2	93	80	173	
3	122	137	259	
4	1	2	3	
Surgery type				< 0.001*
SG	147 (67.1%)	74 (33.8%)	221 (50.5%)	
RYGB	72 (32.9%)	145 (66.2%)	217 (49.5%)	
Pre-op therapeutic anticoagulation	11 (5.0%)	13 (7.0%)	24 (5.9%)	0.417
Platelet inhibition	13 (5.9%)	12 (5.5%)	25 (5.7%)	0.500
Type II diabetes				
NIDDM	53 (24.2%)	49 (22.4%)	102 (23.3%)	0.367
IDDM	24 (11.0%)	22 (10.0%)	46 (10.5%)	0.438
Hypertension	137 (62.6%)	119 (54.3%)	256 (58.4%)	0.050*
Pre op HB (g/dl)	14.1 ± 1.4	13.9 ± 1.3	14.0 ± 1.3	0.051
Volume intraop (ml)	2061 ± 511.7	2086 ± 698.1	2073 ± 611.5	0.334
Blood loss intraop (ml)	33.7 ± 41.8	27.7 ± 29.3	27.7 ± 29.3	0.054

### Bleeding

Bleeding, as defined above (Hb drop ≥ 2.5 mg/dl and at least one of the following factors: lactate ≥ 2 mmol/L, bloody drainage or more than 200 ml or radiological signs of bleeding) occurred in 33 (10%) patients. Overall, a defined bleeding was either managed conservatively (*n* = 26, 78.8%) or with surgery (*n* = 7, 21.2%). Control group A had 22 patients with bleeding, compared to 11 patients in group B. Postoperative bleeding requiring surgical intervention, occurred overall in seven (1.6%) patients, six without intraoperative blood pressure elevation test (Control vs. Intervention: 2.7% vs. 0.5%, Table 2). Out of the 22 patients managed conservatively, 2 received an endoscopy with bleeding from the gastrojejunal junction and 3 patients were given tranexamic acid while withholding prophylactic anticoagulation postoperatively. One patient showed signs of tachycardic arrythmia and was thus given intravenous fluids, medication, and therapeutic anticoagulation to prevent any coagulative disorders such as stroke. As no signs of abdominal bleeding were observed, the drop in hemoglobin was mostly likely due to the application of intravenous fluids. After observation no further HB drop was observed. The rest of the patients (*n* = 16) were closely observed with follow-up hemoglobin controls; no further drop was observed; thus, no further intervention took place. Patients’ demographics and clinical factors for each individual patient requiring surgical intervention are shown in Table 3.

**Table 2:** Bleeding with (group B) and without (group A) surgical intervention across the groups

	Group A (*n* = 219, %)	Group B (*n* = 219, %)	Significance (*p* =)
Bleeding	22 (10%)	11 (5%)	0.034*
No bleeding	197 (90%)	208 (95.0%)	
Surgical revision	6 (2.7%)	1 (0.5%)	0.061
No bleeding	213 (97.3%)	218 (99.5%)

**Table 3:** Individual demographics and clinical factors for patients with bleeding complications requiring surgical intervention

Group A/B	Control A (1)	Control A (2)	Control A (3)	Control A (4)	Control A (5)	Control A (6)	Intervention B (7)
Gender	Female	Female	Female	Male	Female	Male	Male
Type of operation	RYGB	RYGB	RYGB	SG	SG	SG	SG
Age (years)	38	42	63	54	51	41	50
Ther. anticoa-gulation postop (day 1 & 2)	No	No	No	No	No	No	No
Surgery time (min)	137	147	196	95	82	86	70
Hypertension	No	Yes	Yes	Yes	Yes	Yes	Yes
Diabetes	No	Yes	Yes	Yes	No	Yes	Yes
Pre-op BMI (kg/m^2^)	38.9	42.5	44.2	44.1	36.5	51.2	42.2
Preop Hb (g/dl)	13.2	13.9	15.1	14.2	13.3	16.9	16.6
Postop Hb	12.8	13.8	15.0	14.2	13.1	16.0	16.0
Hb day 1	8.9	8.8	11.2	11.5	12.0	15.3	11.8
Hb day 2	6.8	9.3	9.7	9.9	8.5	14.1	10.4
Lactate (mmol/L)							
6 h	1.5	1.1	1.4	1.5	0.7	1.4	1.3
12 h	1.5	2.5	1.9	1.6	0.8	1.4	5.5
Quantity of drain (ml)	300	200	100	500	100	50	600
Quality of drain	Bloody	Bloody	Bloody	Bloody	Bloody	Bloody-serous	Bloody
Postop BP (mmHg)	133/91	125/74	N/a	82/52	139/61	142/82	182/89
Radiological Signs	None	Free fluid in ultra-sound	Free fluid in ultra-sound	None	None	Hema-toma in CT	None
Hours until revision	6	7	22	6	48	60	15
Transfusion	No	2 EKs	2 FFPs	No	2 Eks	No	No
Localization of bleed	Staple line (pouch)	Staple line (pouch)	Fascia edges	Staple line	Staple line	No clear source	Staple line

In multivariate analysis, the intervention shows a significant reduction in bleeding rates (*p* = 0.047). For patients with surgical management of postoperative bleeding multivariate analysis, blood pressure regulation did not show a statistical significance (*p* = 0.057). None of the patients requiring surgical intervention had therapeutic anticoagulation; by default, this is statistically significant (*p* = 0.007). No other significant factors could be identified in multivariate analysis. All factors analyzed in multivariate analysis are depicted in Table 4.

**Table 4:** Multivariate analysis of independent factors for postoperative bleeding

Influencing factors for postoperative bleeding	Significance (*p* =) for all postoperative bleeding (*n* = 22)	Significance (*p* =) for postoperative bleeding with surgical intervention (*n* = 7)
Gender	0.712	0.171
Age	0.219	0.263
BMI preop	0.185	0.124
Hypertension	**0.036**	0.141
Platelet inhibition	0.385	0.325
Therapeutic anticoagulation	0.879	0.007*
Type of surgery	0.338	0.722
Intervention vs. control	0.047*	0.057

## Discussion

This retrospective, observational analysis evaluated whether intraoperative blood pressure increase to 150 mmHg systolic and subsequent hemostasis could decrease the rate of postoperative bleeding after bariatric surgery, without any further intervention regarding staple line reinforcement. In 2015 this intervention was added to our surgical bariatric standard, no randomization or matching was performed. Based on 438 consecutive patients, we have shown a decrease in bleeding rates after introducing intraoperative blood pressure increase from 10% to 5.0% (*n* = 22 vs. *n* = 11). The rate of revisional surgery for postoperative bleeding also decreased from 2.7% to 0.5% (*n* = 6 vs. *n* = 1). Although not statically significant, most likely due to the small sample size, the differences in absolute numbers before and after the intervention of intraoperative blood pressure regulation show a strong effect on bleeding rates for patients needing surgical intervention. In multivariate analysis intraoperative blood pressure regulation and hypertension showed a statistical significance in overall bleeding rates. No statistically significant advantage for the blood pressure regulation test is found for patients who needed a re-operation. None of the patients needing surgical intervention was using therapeutic anticoagulation; thus, the statistical significance is likely to be a possible coincidence.

**Fig. 1:**
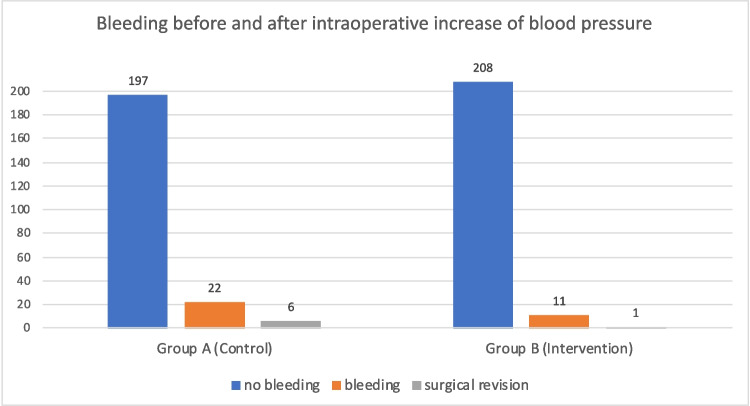
Comparison of bleeding rate before and after the implementation of intraoperative blood pressure increase

Overall, our postoperative bleeding rates are comparable to those quoted in the literature, although no standardized definition was found to define postoperative bleeding [11–23] (Fig. 1). In the clinical setting, bleeding is difficult to define alone on measurements such as hemoglobin drop and lactate levels, as they can easily be influenced by, e.g., the application of intravenous fluids or other underlying comorbidities. Thus, it is usually a combination of quantifiable relevant factors (Hb, lactate level, drain quantity, and quality), clinical signs and symptoms (blood pressure, heart rate, hemodynamic instability, abdominal pain) and radiological signs (presence of free fluid on CT or ultrasound) and level of expertise. The retrospective, individual, and highly clinical diagnosis of bleeding makes rates difficult to compare across the literature. Furthermore, the indication of surgical intervention will differ across different bariatric units. Some surgeons will have a more conservative approach with conservative measures, whereas in our unit all patients with definite signs of acute early bleeding will receive a surgical revision. The indication for surgical revision for hematomas differs again. Here clinical signs such as abdominal pain or signs of infection will play an important role for the indication of revisional surgery. It is, however, possible that some patients with asymptomatic small hematomas were not diagnosed and results might thus be skewed.

Intraoperative blood pressure regulation has been investigated in several studies [12, 29, 31] and is standard procedure since 2015 in our bariatric center. It is part of current recommendations, which include (1) reinforcement of the staple line, (2) choice of adequate staple cartridge, and (3) intraoperative blood pressure control [23]. Evidence of intraoperative blood pressure increase without SLR does not currently exist, compared to multiple studies that evaluated the effectiveness of SLR. Using catecholamines to increase blood pressure intraoperatively could have potential cardiovascular side effects. However, as the blood pressure is only elevated to above 150 mmHg systolic for a short period of time, we have not come across relevant side-effects of this intervention. Nevertheless, patients with high risk of cardiovascular side-effects should be carefully monitored.

Different methods, including oversewing, bovine or polymer membranes, and fibrin glue, have been described, with no definite conclusion in terms of superiority. A meta-analysis by Shikora et al. compared four SLR options and showed decreased bleeding rates with bovine and polymer membrane SLR, and oversewing still showed better outcomes than when no SLR was used [17]. Similarly, Berger et al*.* evaluated almost 200,000 patients with and without SLR and showed SLR to significantly decrease hemorrhagic complications [11]. Although most studies have shown a decrease in postoperative bleeding, not all came to the same conclusion [24, 25]. In a systematic review by Knapps et al., leakage rate could be significantly reduced by reinforcement of the staple line, but no difference could be seen regarding bleeding rates [16]. Due to the conflicting evidence, SLR is not used in our bariatric center. Oversewing the entire staple line can potentially add relevant operating time and should be performed by experienced laparoscopic surgeons. Furthermore, there is a risk of further complications (e.g., leakage or perforation) when oversewing is not done adequately. This is supported by Berger et al.’s study, where patients with SLR showed higher leak rates and only minimally lower bleeding rates (1% vs. 0.75%) [11]. SLR with bovine or polymer membranes have shown some success, however, in our opinion, the additional material costs do not justify the potential small decrease in postoperative bleeding rates. Thus, intraoperative blood pressure increase alone has been the intervention of choice over the last years in our center, as it is uncomplicated, does not infer additional potential complications, and does not add operation time or costs in our opinion. After blood pressure increase, small bleeds can be easily identified along the staple line/gastroomentalis arcade and selectively clipped using titanium clips. No side effects of increased blood pressure or catecholamine use were observed, making it a safe and easy intervention to apply in clinical practice.

Naturally, this observational, retrospective study has its limitations. As these are consecutive patients, no randomization or matching occurred between groups and patients. Group A included patients with higher preoperative BMI and thus potential higher level of difficulty. This could infer higher bleeding rates. The type of surgery differs greatly between the two groups, which could potentially influence our results. Despite a different underlying pathology of bleeding between SG and RYGB, similar rates are quoted in the literature, and can thus be compared. The trend toward more RYGBs over the years was a natural development of our center. This, however, was accounted for in our multivariate analysis. Selection bias exists as bleeding was defined post hoc. There is no standardized definition of postoperative bleeding, making comparison within the literature more difficult. The inhomogeneity on a statistical level is most likely due to small number of patients. For a statistical significance, a higher sample size (*n* > 1000) would be needed, and matching should occur to determine clinical significance more thoroughly.

Overall, the use of increased intraoperative blood pressure alone, without SLR reinforcement, could potentially be an adequate way to reduce post-bariatric hemorrhagic complications (with and without necessary surgical intervention). For this intervention, no specific surgical expertise is required, no additional costs occur, and no relevant further operating time is added, thus minimizing risks for the patient. Due to the retrospective, purely observational nature of this study no definitive conclusion can be reached, without more prospective data with larger sample sizes.
